# Evaluating Disparities in Places of Death in the United States Among Patients With Intellectual Disabilities: A 22-Year Analysis Using the CDC-WONDER Database

**DOI:** 10.7759/cureus.46347

**Published:** 2023-10-02

**Authors:** Raizel Michelina Suresh, Fatima Afzal, Aiah Mounir Abdel-Aal, Siddharth Singla, Aryaa Dixit, Riley Charanrak

**Affiliations:** 1 Internal Medicine, Stanley Medical College, Chennai, IND; 2 Internal Medicine, Dow Medical College, Karachi, PAK; 3 Internal Medicine, Faculty of Medicine, University of Alexandria, Alexandria, EGY; 4 Pathology, Dr. DY Patil Medical College, Pune, IND; 5 Psychiatry and Behavioral Sciences, Mahatma Gandhi Medical College and Research Institute, Puducherry, IND; 6 Internal Medicine, Medical University of Lublin, Lublin, POL

**Keywords:** palliative care, end-of-life care, hospice care, home care, mental retardation, cdc wonder database, mortality trends

## Abstract

Background

In the United States, intellectual disabilities are related to higher death rates. Given the relationship between intellectual disabilities and places of death, it is important to evaluate trends and disparities in places of death to direct physician and patient education and improve the chances of patients who will receive end-of-life care to be suitable with their preferences and values. In this study, the data from the CDC Wide-ranging Online Data for Epidemiologic Research (WONDER) database was used to examine mortality trends in places of death of patients with intellectual disabilities in the United States between 1999 and 2020.

Methodology

The data were collected with the International Classification of Diseases 10th Revision codes F70-F79 (intellectual disability) from the CDC WONDER website on July 20, 2023, to evaluate disparities in places of death. R programming software was used to conduct statistical analysis for predicting death trends in hospice or home care for age groups, genders, census regions, and races.

Results

In total, 9,432 patients with intellectual disabilities died between 1999 and 2020. The highest number of deaths was in the age group of 55-64 years (2,281) and the lowest was in the age group of 1-4 years (55). The data showed that mortality rates among various demographic groups varied significantly. The age group of 25-34 years was more likely to die in a home/hospice setting (odds ratio (OR) = 2.137, confidence interval (CI) = 1.452, 3.145, p < 0.001), considering 85+ years as the reference age. The age group of 1-4 years had the lowest risk of death at home or hospice (OR = 0.147, CI = 0.108, 0.2, p = 0.001).

Conclusions

The deaths from intellectual disabilities from 1999 to 2020 were higher in medical facilities than those in home or hospice care. The mortality was higher for older adults, whites, and males in the Midwest and the South regions, as well as in medical faculties compared to other categories.

## Introduction

Death has always been a sensitive topic to discuss in healthcare because it causes discomfort to patients and their families. However, in many situations, we find it crucial to discuss death and our plan to manage the end-of-life stage to make it easier and more comfortable for the patient. Every person, no matter what their race, sex, or age, deserves to have an equal opportunity to receive the same comfort in their last days [1]. Many patients and their families have different beliefs regarding where they prefer to spend their end-of-life stage. Many prefer to spend it at home or in hospice care so they can be closer to their families than in hospitals or nursing homes [2]. Observing the mortality trends helps patients and their care providers understand the preferences of patients as well as guide healthcare systems to adjust these preferences and provide critically ill patients with what they need to be as comfortable in their last few days as possible.

Intellectual disability is defined as a significant decline in intellectual function associated with substantial limitations in present functioning in adaptive skills such as communication, self-care, home living, social skills, health and safety, leisure, and work. It is also associated with many morbidities that increase the mortality risk by two to four folds compared to the general population [3]. The most common disorders leading to death are respiratory and nervous disorders [4]. Pain and psychological distress are important concerns to be managed as part of palliative management, as are respiratory distress, swallowing problems, and seizures [4]. All these symptoms can be easily managed in hospitals and nursing homes, as well as in hospice facilities and homes. Therefore, it is valuable to understand the disparities in the rate of deaths for patients with intellectual disabilities to be able to provide the best care for their comfort.

This study aims to evaluate the mortality trends and disparities in places of death of patients with intellectual disabilities in the United States.

## Materials and methods

This epidemiological (observational) study was conducted in July 2023. Data were extracted from the CDC Wide-ranging Online Data for Epidemiologic Research (WONDER) database on July 20, 2023. The National Center for Health Statistics provides the national mortality data used in the WONDER database, which is based on records from the death certificates of US citizens [5].

Various parameters were selected to obtain the data. The study period was set as 1999-2020. The underlying causes of death were searched for intellectual disabilities. This was determined using the following International Classification of Diseases, 10th revision (ICD-10) codes: F70 (mild intellectual disability), F71 (moderate intellectual disability), F72 (severe intellectual disability), F73 (profound intellectual disability), F78 (other intellectual disabilities), and F79 (unspecified intellectual disability).

The following four parameters were used to analyze the data: age group (1-85 years old), gender (male or female), census regions of the United States (Northeast, Midwest, South, and West), and race (American Indian or Alaska Native, Asian or Pacific Islander, Black or African American, White) from 1999 to 2020. The place of death was grouped as a medical facility (outpatient, emergency room, inpatient, death on arrival), nursing home, hospice, or others.

This study was exempt from Institutional Review Board review as CDC WONDER is a de-identified and publicly available database. The data was then exported to a Microsoft Excel sheet, where the total deaths for all years based on the age groups, gender, race, and census region according to their place of death were summarized for better understanding. To facilitate further analysis, the data for deaths that occurred while receiving home and hospice care in each category were further divided into each individual year from 1999 to 2020.

Statistical analysis for predictors of death trends in hospice or home care for all groups was done through R programming software using a univariate logic regression model with each group as a variable. Considering a single variable from the group as an initial reference, the odds ratio (OR) and its confidence interval (CI) were calculated for each group. The future death trends until the year 2025 were predicted using the autoregressive integrated moving average model.

## Results

In this study, aggregated data on 9,432 deaths due to intellectual disabilities from 1999 to 2020 was obtained from the CDC WONDER database.

Table 1 illustrates the place of death from mental retardation in the years 1999-2020. The greatest number of deaths were reported in the age group of 55-64 years (2,281). In other places, no deaths due to intellectual disabilities were reported among children aged 1-4 years or 5-14 years. Deaths reported in people with intellectual disabilities were more common in males than females in all places of death. There were more deaths among whites compared to others. Among American Indians and Alaskan Natives, all deaths in the database for the years 1999-2020 were in medical facilities, nursing homes, or long-term care. Among the deaths in homes or hospices, the greatest number of deaths, i.e., 353 deaths, were reported in the age group 55-64 years. In total, 501 deaths were reported from Census Region 3: South.

**Table 1:** Places of death of people with intellectual disabilities in the years 1999-2020. The data was obtained using the CDC WONDER database. The values are mentioned as absolute number N.

	Home or hospice (n = 1,527)	Medical facility or nursing (n = 7,421)	Others (n = 484)
Age groups			
1–4 years	13	42	0
5–14 years	34	124	0
15–24 years	91	279	10
25–34 years	103	445	42
35–44 years	198	777	52
45–54 years	294	1,423	88
55–64 years	353	1,803	125
65–74 years	236	1,292	74
75–84 years	146	883	33
85+ years	47	305	14
Gender			
Female	679	3,207	204
Male	848	4,214	280
Census Region			
Census Region 1: Northeast	319	1,438	62
Census Region 2: Midwest	457	2,262	129
Census Region 3: South	501	2,544	164
Census Region 4: West	250	1,173	119
Race			
American Indian or Alaska Native	0	10	0
Asian or Pacific Islander	14	74	0
Black or African American	194	930	62
White	1,308	6,402	413

Table 2 highlights the predictors of home or hospice death in people with intellectual disabilities in the years 1999-2020 using values obtained from the CDC WONDER database. According to univariate logistic regression, and considering 85+ years as the reference age, the age group of 25-34 years was more likely to die in a home or hospice setting (OR = 2.137, CI = 1.452, 3.145, p < 0.001). The age group of 1-4 years was least likely to die in the home or hospice setting (OR = 0.147, CI = 0.108, 0.2, p < 0.001). Among census regions, considering Census Region 1: Northeast as the reference, people with intellectual disabilities in Census Region 3: South were less likely to die in a home or hospice setting (OR = 0.87, CI = 0.746, 1.015, p = 0.076).

**Table 2:** Predictors of home or hospice death in people with intellectual disabilities in the years 1999-2020 using values obtained from the CDC WONDER database.

Variables	Univariate logistic regression		
	Odds ratio	95% confidence interval	P-value
Age			
1–4 years	0.147	(0.108, 0.2)	< 0.001
5–14 years	2.101	(1.05, 4.202)	0.036
15–24 years	1.861	(1.143, 3.03)	0.013
25–34 years	2.137	(1.452, 3.145)	< 0.001
35–44 years	1.435	(0.989, 2.084)	0.057
45–54 years	1.621	(1.15, 2.285)	0.006
55–64 years	1.321	(0.949, 1.838)	0.099
65–74 years	1.243	(0.896, 1.723)	0.192
75–84 years	1.173	(0.838, 1.641)	0.353
85+ years	1.0 (Reference)		
Gender			
Male	1.000 (Reference)		
Female	1.055	(0.945, 1.178)	0.342
Census Region			
Census Region 1: Northeast	1.000 (Reference)		
Census Region 2: Midwest	0.899	(0.768, 1.051)	0.182
Census Region 3: South	0.87	(0.746, 1.015)	0.076
Census Region 4: West	0.91	(0.759, 1.091)	0.308
Race			
American Indian or Alaska Native	0	(0, 0.000)	0.944
Asian or Pacific Islander	0.986	(0.555, 1.751)	0.961
Black or African American	1.019	(0.864, 1.202)	0.823
White	1.000 (Reference)		

**Figure 1:**
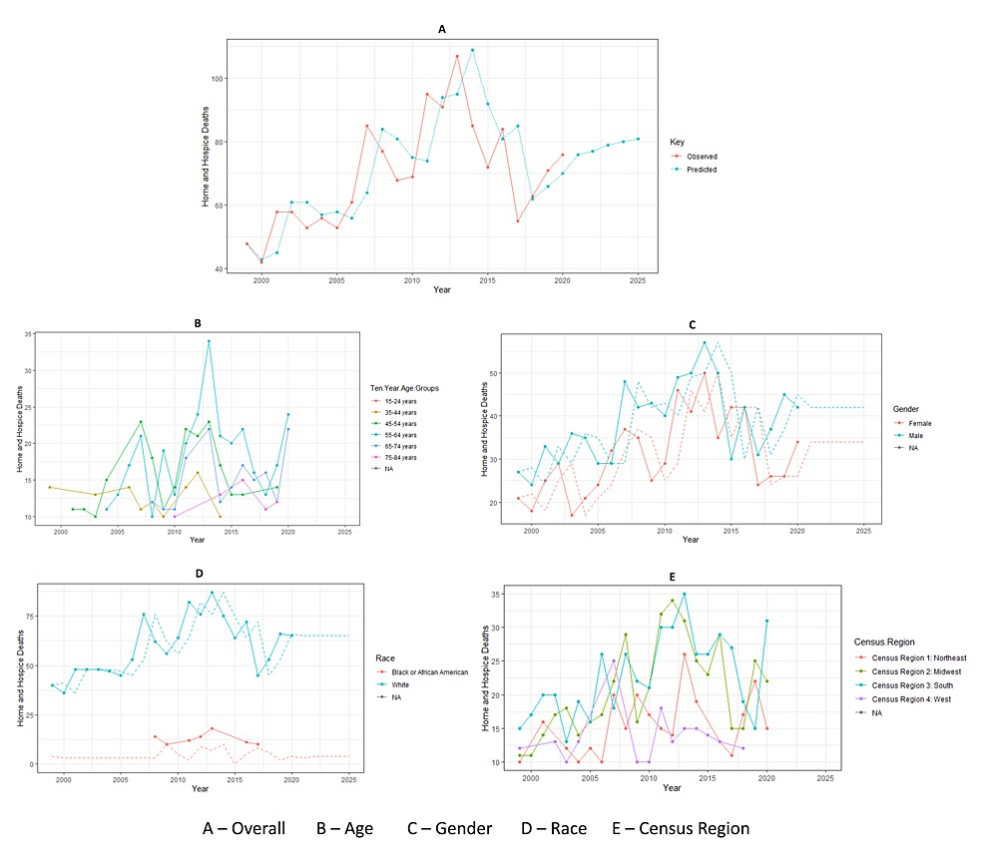
Death trends in the home/hospice setting.

The overall deaths among people with intellectual disabilities in the home or hospice setting showed a trend of steep rises and sudden falls (Figure 1a). The first significant peak was around the year 2007 and then fell suddenly around 2009. The highest number of deaths in the home or hospice was around the year 2013. The lowest number of deaths was in 2005. The most recent sudden fall was around the year 2017, and since then, the overall number of deaths in the home or hospice showed a gradually increasing trend (Figure 1a). The trends observed and predicted are almost similar (Figures 1a, 1c, 1d). Figure 1b and Figure 1e highlight deaths due to intellectual disabilities according to age group and census region. The lowest number of deaths in the home or hospice in the 55-64 age group was in 2008. The most recent low number of deaths for this age group was around 2018. Around 2013, the deaths in the 55-64 age group and the Census Regions 1 and 3 (Northeast and South) increased the highest. In 2020, there was a sudden rise in deaths in the home or hospice setting, both in the 55-64 age group and in Census Region 3: South.

## Discussion

To study the mortality trends of intellectual disabilities, 22 years of data were collected from the CDC WONDER database [5]. First, this study analyzed four parameters, namely, age groups, gender, census region, and race. This study found that there were more deaths due to intellectual disabilities in medical or nursing facilities than in homes or hospices, with 7,421 deaths occurring in medical or nursing facilities and 1,527 deaths in home or hospice settings. These findings are consistent with another exploratory study done in Australasia [6], which found that the majority of deaths in individuals with intellectual disabilities occurred within a hospital setting. There could be many plausible explanations for this. First, patients who suffer from intellectual disability have a higher risk of developing other pathologies such as cardiac problems, pneumonitis, influenza, and diabetes, among others [7]. This could result in higher hospitalization rates, leading to an increase in hospital-related deaths. In this study, 484 deaths that did not fit the categories of home/hospice care or hospital/nursing facility were included under the other category. Furthermore, in the category of 10-year age groups, the highest number of deaths were reported in the 55-64-year age group in both hospital/nursing facilities and home/hospice care (1,803 and 353 deaths, respectively). Studies have shown the average age of death in people with intellectual disabilities to be in the late 50s and 60s [8,9]. Overall, a decrease in age-at-death disparity between adults with and without intellectual disabilities has been seen over the years [9] due to improvements in health and easier access to medical care. This study also showed that the 1-4-year age group had the lowest mortality rates among all the age groups. This can be explained by the fact that the most prevalent form of intellectual disability is mild intellectual disability, according to the American Psychiatric Association [10], and children with mild intellectual disability are more likely to have significantly lower mortality rates.

Moreover, there were more deaths from intellectual disabilities in males compared to females in home/hospice and medical/nursing facilities (848 deaths vs. 679 deaths in home/hospice and 4,214 vs. 3207 deaths in hospital/nursing facilities). There is evidence that the prevalence of intellectual disability is higher among boys compared to girls [11]. X-linked disorders, such as fragile X syndrome, could be one of the reasons for this [12]. A higher prevalence can then explain a higher mortality rate in males. A study in Ontario also showed a higher rate of mortality among men in the age group of 25-99 years [13].

Among the four census regions, the South census region had the most deaths, i.e., 501 and 2,544 deaths in home/hospice and hospital/nursing facilities, respectively. The findings of a study on the state-specific rates of intellectual disabilities [14] closely match our findings. According to this study, the highest overall rates of intellectual disabilities were in the East South Central, South Atlantic, and West South Central regions. The South Census Region includes all these regions, according to the current CDC guidelines [15]. The lowest number of deaths was seen in the West census region. Plausible explanations include the better socioeconomic status of people in this region, though further studies are warranted in this regard.

Lastly, within the racial groups, the highest number of deaths were reported in the white population, with 6,402 deaths in hospital or nursing facilities and 1,308 in home or hospice care. This can be explained by the fact that the majority of the US population is white. The lowest number of deaths was reported among American Indians or Alaska Natives, with no deaths reported in our study in hospice settings and 10 deaths in hospital or nursing facilities. While this can also be attributed to the fact that this group makes up a lower percentage of the US population, studies have shown significant racial disparities in hospice care, with minorities using these services less compared to whites [16]. A literature review on palliative care for American Indians and Alaska Natives also showed that the utilization of hospice services was lower among this racial group [17], which is consistent with our findings.

It is routinely observed that patients with intellectual disabilities and other comorbidities are often dependents who are subjected to various socioeconomic factors such as social neglect, isolation, discrimination, and poverty, rendering them unable to avail themselves of, access, or communicate their health needs and requirements in a professional healthcare setup.

Limitations

This study has a few limitations. First, the data from 2021 to 2023 was excluded because it was not included in the CDC WONDER database. Second, while this study evaluates mortality trends among all categories of intellectual disability (F70-F79), it fails to evaluate mortality trends within the subcategories of mild (F70), moderate (F71), severe (F72), profound (F73), other (F78), and unspecified (F79). To determine more precise patterns, additional research can be done to examine mortality trends within each of these subcategories.

## Conclusions

This study concluded that people in the 10-year age group, i.e., 55-64 years, males, the South Census Region, and the white population had the highest death rates in hospice and home care compared to other subgroups. According to this study, the age group of 25-34 is more likely to die in home or hospice settings. The overall number of deaths in the home and hospital settings also showed a gradually increasing trend. Further research is needed to study the reasons for these findings and establish trends to achieve significant improvements in end-of-life care. For instance, this study showed that people in the South census region are less likely to die in home/hospice settings. Additional research is required to understand the reason for this trend, how to apply that knowledge, and how it can help improve patient comfort. With the rapid advancements in healthcare, it is pertinent that measures are taken to ensure patients can have a *good death* with *improved quality of dying*. It is also important to acknowledge the additional challenges of providing palliative care to individuals with intellectual disabilities. This includes difficulty in communication between patients and care providers, as well as a lack of proper training and education about providing palliative care to patients with intellectual disabilities. By carrying out further research, it will be possible to identify mortality trends in more depth, and this information can then be used to improve palliative care.
